# Fibrilação Atrial (Parte 2) – Ablação por Cateter

**DOI:** 10.36660/abc.20200477

**Published:** 2021-02-19

**Authors:** Eduardo B. Saad, Andre d’Avila

**Affiliations:** 1 Hospital Pró-Cardíaco Serviço de Arritmias e Estimulação Cardíaca Artificial Rio de JaneiroRJ Brasil Hospital Pró-Cardíaco - Serviço de Arritmias e Estimulação Cardíaca Artificial, Rio de Janeiro, RJ - Brasil; 2 Hospital Samaritano Rio de JaneiroRJ Brasil Hospital Samaritano, Rio de Janeiro, RJ - Brasil; 3 Hospital SOS Cardio FlorianópolisSC Brasil Hospital SOS Cardio, Florianópolis, SC - Brasil; 4 Beth Israel Deaconess Hospital Harvard Medical School Boston EUA Beth Israel Deaconess Hospital, Harvard Medical School, Boston - EUA

**Keywords:** Arritmias Cardíacas, Fibrilação Atrial/terapia, Ablação por Cateter/métodos, Ecocardiografia/métodos

## Abstract

Após mais de 20 anos desde sua utilização inicial, a ablação por cateter se tornou um procedimento rotineiramente realizado para tratamento de pacientes com fibrilação atrial (FA). Fundamentado inicialmente no isolamento elétrico das veias pulmonares em pacientes com FA paroxística, subsequentes avanços no entendimento da fisiopatologia levaram a técnicas adicionais não só para obter melhores resultados, mas também para tratar pacientes com formas persistentes de arritmia, assim como pacientes com cardiopatia estrutural e insuficiência cardíaca.

Significativos avanços tecnológicos, em especial no mapeamento eletroanatômico 3D, no uso de ecocardiograma intracardíaco e na forma de energia aplicada (crioablação e força de contato tecidual com radiofrequência), permitiram redução significativa na taxa de complicações e no uso de radiação ionizante.

Atualmente, a ablação é o tratamento mais eficiente para pacientes com FA, sendo uma excelente alternativa ao uso de fármacos antiarrítmicos, cujo desenvolvimento foi insignificante nas últimas décadas.

Com as pioneiras observações feitas por Haissaguerre et al.,^1^ demostrou-se o papel fundamental de focos arritmogênicos nas veias pulmonares (VP) na fisiopatologia da iniciação e na manutenção dos episódios de FA. Nestas foi estabelecido o conceito de FA focal, em que uma arritmia atrial que acomete difusamente ambos os átrios tem origem bem determinada, logo, passível de intervenções terapêuticas. Técnicas utilizando a ablação por cateter foram desenvolvidas e aperfeiçoadas, visando à eliminação dos focos geradores da FA por meio da ablação circunferencial ao redor das VP,^2 - 4^ com índices de sucesso e *performance* superiores quando comparados com a melhor terapêutica farmacológica.^5 - 10^

O objetivo deste artigo é revisar os avanços ocorridos no tratamento ablativo da FA e descrever para o cardiologista clínico o atual estado da arte em relação a suas indicações, técnicas, resultados e complicações.

## Estratégias de ablação

**Figura 1:**
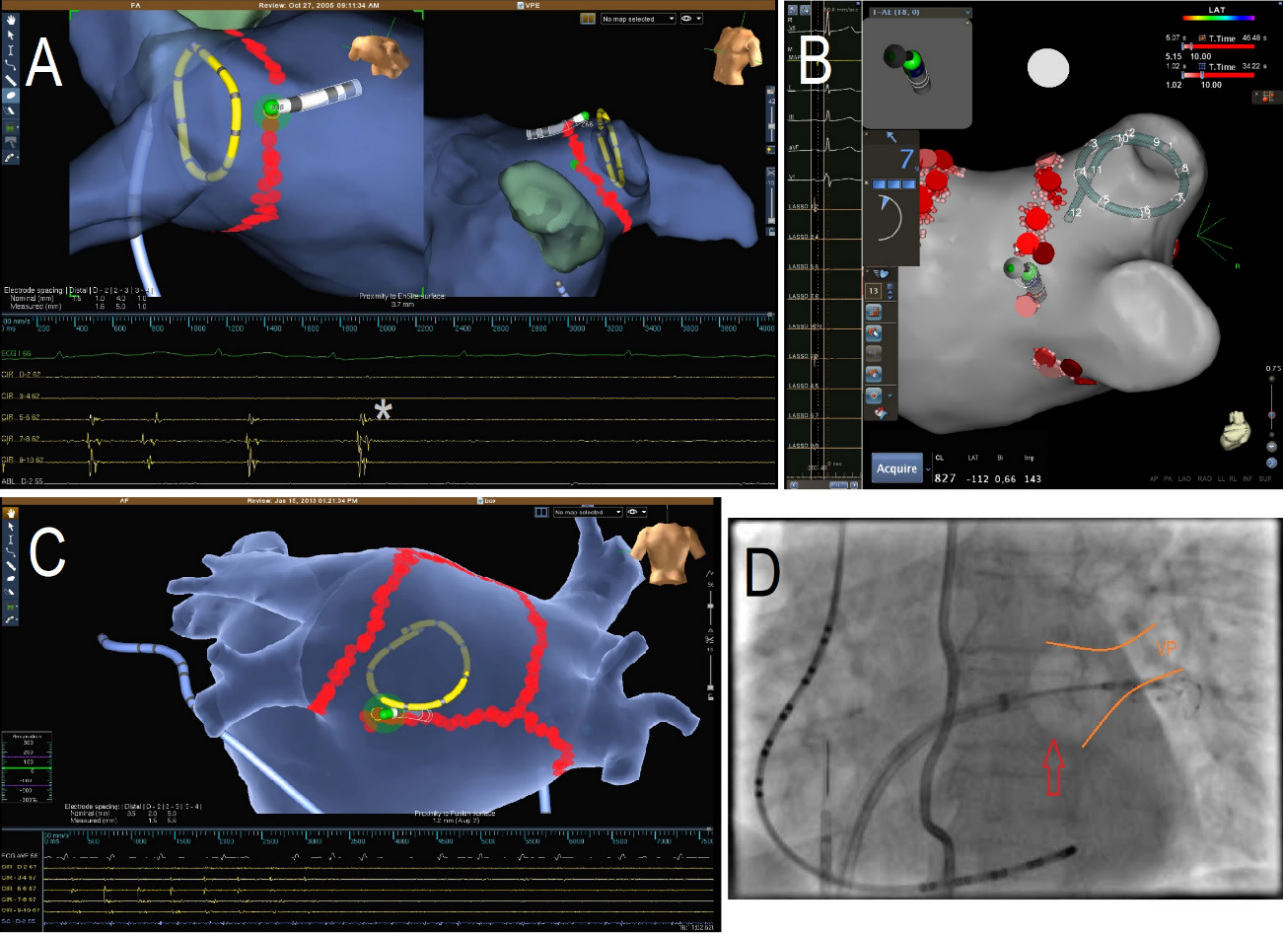
Ablação por cateter para tratamento da FA paroxística. A) Isolamento das VP esquerdas por ablação circunferencial (RF ponto a ponto) guiado por mapeamento eletroanatômico 3D (sistema NAVx – Abbott), demonstrando o desaparecimento dos eletrogramas (*) registrados por um cateter circular no interior das VP. B) Isolamento das VP direitas (sistema CARTO – Biosense Webster) com cateter com sensor de contato tecidual (demonstrado pelo vetor de força e pela quantificação = 7g); o cateter circular de mapeamento está no interior da VP superior direita. C) Ablação de FA persistente (sistema NAVx – Abbott) demonstrando a extensão das lesões de RF para isolamento da parede posterior do AE (lesão linear no teto e na parte inferior), levando ao desaparecimento dos eletrogramas na parede posterior (registrados pelo cateter circular de mapeamento). D) Imagem fluoroscópica durante crioablação para isolamento da VP superior esquerda, demonstrando o cateter-balão (seta) insuflado e em contato com o óstio dessa veia. A ablação em toda a circunferência da VP é realizada simultaneamente pelo balão, que usualmente é restrito para isolamento das VP – quando necessário ablação adicional, um cateter de RF deve ser utilizado.

Ao longo dos últimos 20 anos, diversas estratégias de ablação foram utilizadas para controle da FA. Em comum, é consenso atual que o isolamento de todas as VP é fundamental em todos os grupos de pacientes (FA paroxística, persistente ou persistente de longa duração).^11 - 15^ Esse isolamento deve ser comprovado eletricamente por mapeamento circular no interior das VP (Figuras 1 e 2), pois essa etapa é primordial para o sucesso do procedimento. Estudos recentes mostram que deve, inclusive, ser realizado sem interrupção da anticoagulação oral, estratégia que reduz os riscos trombóticos e hemorrágicos.^16 - 18^

Em pacientes com FA paroxística, em geral, o isolamento das VP é tudo que é necessário, realizando-se lesões adicionais apenas em situações específicas (p. ex., focos deflagradores mapeados fora das VP). Alguns centros realizam de rotina o isolamento da veia cava superior,^19 , 20^ visto que também pode ser, mais raramente, *fonte deflagradora de ectopias e arritmias* , podendo induzir FA. A maioria das publicações demonstra resultados favoráveis, com taxas de sucesso superiores a 70%.^6 , 7 , 9^

O isolamento das VP pode ser realizado utilizando: 1) energia de radiofrequência (RF), por meio de aplicações focais ponto a ponto (Figura 1A), idealmente com cateteres com sensores da pressão tecidual exercida ( Figura 1 B), ou 2) congelamento (crioablação), utilizando um cateter-balão posicionado no antro das VP, capaz de realizar toda a lesão tecidual simultaneamente na circunferência em contato com o tecido (Figura 1D). Estudo randomizado (Fire and ICE)^21^ comparando diretamente as duas estratégias para tratamento de FA paroxística demonstrou resultados semelhantes. Esses achados foram replicados em um segundo estudo randomizado (CIRCA-DOSE)^22^ que comparou dois regimes de crioablação (4min *vs* . 2min de congelamento) ao uso da RF com força de contato para isolamento das VP em pacientes com FA paroxística. Nesse estudo, observou-se redução da carga de FA >98% por meio de monitoramento eletrocardiográfico contínuo. Importante salientar que o cateter-balão usualmente não é utilizado para ablação em outras regiões além das VP; quando necessário, deve-se utilizar um cateter de RF (Figura 1C).

Já nas formas persistente e persistente de longa duração, a criação de barreiras elétricas adicionas é frequentemente realizada, visto que o simples isolamento das VP é insuficiente e associado a altas taxas de recorrência.^23 - 25^ Diversas estratégias foram estudadas,^26 - 37^ sendo as mais frequentemente utilizadas: ablação de deflagradores fora das veias pulmonares, lesões lineares no átrio esquerdo (AE) e extensas aplicações de RF em sítios com registro de eletrogramas fracionados durante a FA (mais comumente observados na parede posterior, septo, teto, anel mitral, base do apêndice atrial esquerdo (AAE) e no interior do seio coronário). *Durante a aplicação de RF nesses locais,* pode ocorrer reversão a taquicardias atriais regulares ou até mesmo ao ritmo sinusal.

Merece ressalva o resultado negativo do estudo randomizado Star AF II,^38^ que comparou a adição de lesões lineares e ablação de potenciais fragmentados ao isolamento das VP em pacientes com FA persistente. Nesse estudo, não houve diferença na manutenção do ritmo sinusal aos 18 meses entre os grupos (59% para o isolamento apenas *vs.* 49% e 46% nos outros grupos, sem significância estatística). Por isso, muitos centros ainda praticam apenas o isolamento das VP mesmo em pacientes com FA persistente.

Uma estratégia mais agressiva para eliminação de deflagradores foi também testada em um estudo randomizado controlado (BELIEF Trial)^39^ – o isolamento elétrico do AAE. Neste, o isolamento dessa estrutura em adição à ablação convencional foi associado à redução de 55% no risco relativo de recorrência de FA em uma população de pacientes com FA *persistente de longa duração* . O isolamento do AAE é atualmente realizado seletivamente devido ao fato de exigir extensas aplicações de RF e adicionar risco para fenômenos embólicos – a perda da contração do AAE leva à remora e formação de trombo. Pacientes com AAE eletricamente isolado devem ser anticoagulados cronicamente independente do escore CHADSVASC, e devem ser submetidos à oclusão dessa estrutura caso o anticoagulante seja contraindicado.^40^

Portanto, em formas mais persistentes de FA com significativo remodelamento atrial, há necessidade de mudança do substrato atrial, implicando maior número e extensão das aplicações de RF. Não há consenso na literatura quanto a melhor estratégia a ser utilizada ( Tabela 1 ). A evolução da FA para formas persistentes representa progressão de um processo patológico^41 , 42^ e deve servir de motivação para o tratamento mais precoce, quando ainda paroxística. Um grande estudo retrospectivo com mais de 4.500 pacientes analisou o impacto do tempo entre o diagnóstico de FA e a realização da ablação.^43^ Os resultados são contundentes, demonstrando que quanto mais cedo a ablação é realizada, melhores são os resultados – estabelecendo o “conceito oncológico da FA”, ou seja, os melhores resultados são obtidos quando se faz o tratamento nas fases iniciais da doença (isolamento das VP em pacientes com FA paroxística). Em fases mais avançadas (FA persistente e permanente), o tratamento é bem mais extenso e com resultados inferiores. Por isso, aqui também, quanto mais cedo, melhor.

**Tabela 1:** – Estratégias durante ablação da fibrilação atrial

Para o isolamento elétrico das veias pulmonares	
Classe I – A	Isolamento das VP é recomendado para todos os procedimentos de ablação de FA
Classe I – B	Demonstração de bloqueio de entrada nas VP
Classe IIa – B	Monitoramento para reconexão das VP por 20 minutos após o isolamento inicial
Classe IIb – B	Administração de adenosina 20min após isolamento das VP Estimulação ao longo da linha de ablação circunferencial Demonstração do bloqueio de saída nas VP
**Em conjunto com isolamento das veias pulmonares**	
Classe I – B	Ablação do ICT em pacientes com histórico de *flutter* típico ou se a arritmia é indutível durante a ablação da FA
Classe I – C	Se forem realizadas lesões lineares, o bloqueio bidirecional deve ser demonstrado
Classe IIa – C	Se forem identificados gatilhos reprodutíveis extra-VP, a ablação deve ser considerada Ao usar um cateter de RF com sensor de contato, um mínimo de 5 a 10g de força é razoável
Classe IIb – B	O isolamento da parede posterior do AE pode ser considerado para ablação inicial ou repetida de AF persistente ou persistente de longa duração. Isoproterenol em altas doses para detecção e ablação de gatilhos extra-VP pode ser considerado para procedimentos iniciais ou reintervenções para FA paroxística, persistente ou persistente de longa duração

*VP: veia pulmonar; FA: fibrilação atrial; RF: radiofrequência; ICT: istmo cavotricuspídeo; AE: átrio esquerdo.*

## Tecnologias para guiar a ablação

Independentemente da estratégia utilizada, métodos de mapeamento por imagem frequentemente são utilizados em adição ao tradicional mapeamento eletrofisiológico. Dois tipos de tecnologia são apropriados nesta circunstância:

- Mapeamento eletroanatômico: esta forma de mapeamento em 3D permite definir com precisão a anatomia da cavidade atrial esquerda e das VP, delinear o substrato funcional pela medida da voltagem tecidual, marcar as lesões de RF ( Figura 1 ) no mapa formado e traduzir em cores a informação elétrica obtida. É possível, também, navegar em imagens da anatomia real originada em tomografia computadorizada ou ressonância magnética. Essa metodologia é especialmente útil para reduzir o tempo de exposição à fluoroscopia, tornando de fácil apreciação o circuito ou o foco da arritmia e as lesões realizadas para tratá-las. Dois sistemas estão atualmente disponíveis no Brasil: *CARTO – Biosense Webster* e *NavX – Abbott Medical* .
- Ecocardiograma intracardíaco (ICE): através de um cateter inicialmente posicionado no átrio direito, é possível obter imagens ultrassonográficas detalhadas da anatomia cardíaca em tempo real,^44 , 45^ permitindo uma manipulação precisa e segura dos cateteres através das diversas estruturas visualizadas ( Figura 2 ). Sua utilização permite também a realização segura das punções transeptais sob visualização direta e a detecção precoce de complicações agudas (derrame pericárdico, trombos). Estudo recente com mais de 100.000 pacientes submetidos à ablação mostra a importância desse método na significativa redução do risco de uma complicação grave – a perfuração cardíaca.^46^ Nessa série contemporânea, o não uso do ICE foi o maior fator de risco para perfuração (RR 4.85).
  **Figura 2:**
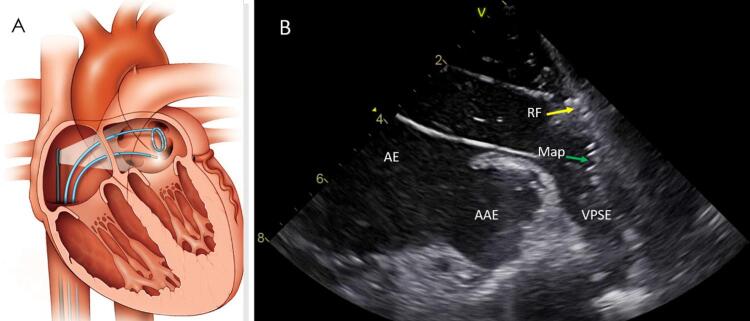
Uso do eco intracardíaco (ICE) durante ablação da FA. A) Desenho esquemático mostrando o cateter de ICE na cavidade atrial direita com feixe direcionado para guiar as duas punções transeptais e o posicionamento dos cateteres de mapeamento circular e de ablação no AE. B) Imagem do ICE demonstrando o posicionamento antral e o contato tecidual durante aplicação de RF ao redor da VP superior esquerda (VPSE). AE: átrio esquerdo; AAE: apêndice atrial esquerdo; Map: cateter de mapeamento; RF: cateter de ablação.

Essas ferramentas não fluoroscópicas têm sido cada vez mais utilizadas nos laboratórios de eletrofisiologia ao longo dos anos e podem até orientar todo o procedimento de ablação, evitando completamente o uso de raios X.^47^ Relatado inicialmente há aproximadamente 10 anos, as técnicas “Zero-Fluoro” estão ganhando popularidade na comunidade eletrofisiológica, porque foram demonstradas tão seguras e eficazes quanto os métodos tradicionais guiados pela fluoroscopia.^48 - 50^

## Recorrências

Dois fatores predominantes justificam as recorrências da FA após ablação:

1. Reconexão da condução nas VP: para que as lesões circunferenciais sejam permanentes, deve ocorrer formação de tecido fibroso, usualmente 4 a 8 semanas após a lesão aguda (edema tecidual). Caso a lesão não tenha profundidade suficiente, pode haver tecido viável remanescente após a reabsorção do edema. Basta um pequeno segmento para restabelecer a condução elétrica.
2. Ocorrência de focos independentes, fora das VP: estes ocorrem mais frequentemente, mas não exclusivamente, em formas persistentes de FA ou em pacientes com remodelamento atrial.

A reconexão das VP é facilmente resolvida com novas aplicações de RF nos *gaps* de condução. A reintervenção geralmente é rápida, fácil e segura. Em FA paroxística, eleva os índices de controle da FA a 95%. Com o uso de cateteres com sensor de contato tecidual, é um fenômeno cada vez mais raro,^51 - 53^ pois as lesões de RF tendem a ser mais profundas e permanentes.^54^

Os focos extra-VP representam um substrato atrial mais difuso, sendo necessários o reconhecimento e a ablação extensa, sem os quais não é possível o controle da arritmia.^33 , 54 , 55^ Mais comumente, envolvem a parede posterior do AE, o AAE e o seio coronário^32 , 54^ – estruturas que podem ser também isoladas por aplicações de RF. É possível a manutenção do ritmo sinusal a longo prazo, mesmo se necessário mais de uma intervenção.

## Seleção de pacientes e resultados

A seleção de pacientes para ablação por cateter da FA baseia-se atualmente na falência do tratamento clínico ( Tabela 2 ). De acordo com o último consenso de especialistas HRS/EHRA/ECAS/APHRS/SOLAECE, de 2017,^11^ a indicação primária para ablação de FA é a presença de fibrilação atrial paroxística ou persistente sintomática, refratária ou intolerante a pelo menos um fármaco antiarrítmico da classe I ou III. Há sólidas evidências de melhora em parâmetros de qualidade de vida desses pacientes.^5 , 56^

**Tabela 2:** – Indicações para ablação por cateter na fibrilação atrial

FA sintomática, refratária ou intolerante a pelo menos 1 fármaco antiarrítmico (classe I ou III)	
Classe I – A	FA paroxística
Classe IIa – B	FA persistente
Classe IIb – C	FA persistente de longa duração
**FA sintomática, antes do início de fármacos antiarrítmicos (Classe I ou III)**	
Classe IIa – B	FA paroxística
Classe IIa – C	FA persistente
Classe IIb – C	FA persistente de longa duração
**Indicações em populações de pacientes pouco representadas em ensaios clínicos**	
Classe IIa – B	Insuficiência cardíaca congestiva Pacientes ≥75 anos de idade Cardiomiopatia hipertrófica Jovens (≤45 anos de idade) Síndrome taqui-bradi
Classe IIa – C	Atletas com FA
Classe IIb – C	FA assintomática

*FA: fibrilação atrial.*

Esta técnica pode ser utilizada em pacientes com diversos tipos de cardiopatia (doença arterial coronária, hipertrofia ventricular esquerda, insuficiência cardíaca) e apresentações clínicas de FA (paroxística, persistente ou de longa duração), porém, os melhores resultados são obtidos nos pacientes com coração estruturalmente normal. No maior estudo randomizado que comparou ablação com tratamento farmacológico (CABANA),^7^ sobrevida livre de recorrência da FA é significativamente melhor (HR 0.53) nos pacientes ablacionados quando comparados aos que permanecem em uso de múltiplos fármacos antiarrítmicos. Apesar disso, nesse estudo, não foi demonstrada redução em desfechos duros combinados (morte, acidente vascular cerebral [AVC], sangramento grave ou parada cardíaca) na análise *intention to treat* , apesar de haver problemas com grande *crossover* de pacientes para o grupo ablação (27%). Nesse estudo, os subgrupos que mais se beneficiaram foram os mais jovens (<65 anos) e os pacientes com insuficiência cardíaca congestiva.

A seleção de pacientes com formas persistente e permanente de FA segue o mesmo raciocínio, porém a decisão deve ser individualizada de acordo com o tamanho do AE^57^(que é um importante fator preditor de recorrência) e a duração da FA. FA persistente é uma doença heterogênea, com diferentes graus de fibrose atrial e com influência do sistema nervoso autônomo e outros processos fisiopatológicos ainda mal compreendidos, o que explica os resultados heterogêneos observados com diferentes estratégias de ablação. Essa forma exige uma definição individual do substrato e mecanismos envolvidos.^58 , 59^

É importante notar que, mesmo com a estratégia de extensas aplicações de RF descritas, o índice de recidivas e a necessidade de novos procedimentos são maiores. Na experiência de Natale et al., 60% dos pacientes mantiveram ritmo sinusal sem fármacos após o primeiro procedimento.^54^ Naqueles que foram submetidos a uma segunda intervenção, 80% mantiveram ritmo sinusal. A Tabela 3 sumariza alguns dos principais estudos publicados.

**Tabela 3:** – Ensaios clínicos em ablação de fibrilação atrial

Estudo (ano)	Tipo	Estratégia de ablação	Nº	*Follow* -up (meses)	Manutenção do ritmo sinusal	p-valor
**FA paroxística**						
Thermocool AF (2010)	Randomizado ablação RF ou DAA; multicêntrico	IVP CFAE e linhas opcionais	167	12	66%/16%	<0,001
STOP AF (2013)	Randomizado para crioablação ou DAA; multicêntrico	IVP	245	12	70%/7%	<0,001
SMART AF (2014)	Não randomizado; cateter sensor de contato; multicêntrico	IVP CFAE e linhas opcionais	172	12	72,5%/NA	<0,0001
TOCCASTAR (2015)	Randomizado com ou sem cateter sensor de contato; multicêntrico	IVP CFAE, gatilhos e linhas extra-VP opcionais	300	12	67,8%/69,4%	0,0073*
RAAFT-2 (2014)	Randomizado ablação RF ou DAA (primeira linha); multicêntrico	IVP gatilhos extra-VP opcionais	127	24	45%/28%	0,02
MANTRA-PAF (2012)	Randomizado ablação RF ou DAA (primeira linha); multicêntrico	PVI + linha teto linhas mitral e ICT opcionais	294	24	Carga de FA: 13%/19%	-
FIRE and ICE (2016)	Randomizado RF ou crioablação; multicêntrico	IVP	762	12	64,1% (RF)/65,4% (crio)	-
CIRCA DOSE (2019)	Randomizado RF ou crio 4min ou crio 2min; multicêntrico	IVP	346	12	53,9% (RF)/52,2% (crio 4min)/51,7% (crio 2min)	0,87
**FA persistente**						
TTOP (2014)	Randomizado ablação RF ou DAA / CVE	IVP + CFAE	210	6	56%/26%	<0,001
SARA (2014)	Randomizado ablação RF ou DAA; multicêntrico	IVP CFAE e linhas opcionais	146	12	70%/44%	0,002
STAR AF II (2015)	Randomizado 3 estratégias de ablação RF; multicêntrico	IVP; IVP + CFAE; IVP + Linhas	589	18	59%/49%/46%	0,15
**FA paroxística ou persistente**						
CABANA (2019)	Randomizado ablação RF ou DAA; multicêntrico	IVP ablação adicional opcional	2204	48,5	69%/50%	-

FA: fibrilação atrial; RF: radiofrequência; DAA: drogas antiarrítmicas; IVP: isolamento das veias pulmonares; CFAE: eletrogramas atriais fracionados complexos; CVE: cardioversão elétrica; *não inferioridade.

A ablação por cateter é menos eficaz em determinados subgrupos de pacientes,^60^ em que ainda é necessário avançar no conhecimento fisiopatológico: átrios dilatados e fibrosados, FA persistente ou de longa duração, cardiomiopatia hipertrófica, infiltrado amiloide, obesidade e apneia do sono.

O acompanhamento a longo prazo de pacientes submetidos à ablação por cateter mostra que há a possibilidade de recidivas tardias,^61 - 63^ na ordem de 7% ao ano nos primeiros 5 anos. Deve-se destacar que a estimativa do real sucesso da ablação é dificultada pelas inconsistências e heterogeneidades nas definições de sucesso e recorrências nos diferentes estudos. Como exemplo, a maior parte dos estudos considera recidiva qualquer arritmia atrial com duração maior que 30 segundos, uma definição claramente com pouco significado clínico. Nesse cenário, a carga de FA deve ser mais valorizada nas pesquisas futuras.

Com a tendência ao isolamento permanente das VP, observa-se mais frequentemente a recorrência por aparecimento de focos extra-VP, que devem ser identificados e ablacionados.^32 , 33 , 54 , 64^ Portanto, é importante manter a vigilância com monitoramento periódico dos pacientes, sendo prudente manter a terapia anticoagulante nos pacientes de mais alto risco que não apresentem contraindicações.

A Tabela 4 resume os cuidados adjuvantes para maximizar a segurança e a eficácia do procedimento.

**Tabela 4:** – Estratégias adjuvantes à ablação de fibrilação atrial

Não diretamente relacionado ao procedimento de ablação	
Classe IIa – B	Perda de peso Considerar o IMC do paciente na avaliação para procedimento de ablação Pesquisar sinais e sintomas de apneia do sono Tratar apneia do sono
Classe IIb – C	Interrupção de DAA antes da ablação para melhorar os resultados a longo prazo não é clara Uso de DAA durante o período de cicatrização (3 meses) após a ablação para melhorar os resultados não é claro
Redução do risco durante o procedimento de ablação	
Classe I – B	Delineamento preciso da anatomia das VP para evitar ablação no seu interior
Classe I – C	Redução da potência aplicada na parede posterior do AE, próximo ao esôfago
Classe IIa – C	Utilização de um cateter com sensor de temperatura no lúmen esofágico para guiar a titulação da energia aplicada

*DAA: drogas antiarrítmicas; IMC: índice de massa corporal; VP: veia pulmonar; AE: átrio esquerdo.*

## Situações especiais

Diretrizes internacionais publicadas em 2016 e 2017 e atualizadas em 2019 e 2020 pelas diferentes sociedades internacionais (SBC/HRS/EHRA/ECAS/APHRS/ACC/AHA/ESC/EHRA)^11 - 13 , 15^ recomendam de forma quase consensual o tratamento ablativo em situações especiais ( Tabela 2 ):

### 1) Ablação como primeira escolha:

A crescente segurança e a eficácia permitem que a ablação seja oferecida como terapia de primeira linha para tratamento (antes mesmo do uso de fármacos antiarrítmicos) em algumas situações especiais (atletas, jovens, corações normais).^65 , 66^ Em pacientes com FA paroxística ou persistente sintomática é indicação Classe IIa. Situações apropriadas para esta estratégia são pacientes com pausas sintomáticas na reversão (síndrome de taquibradi)67 ou atletas competitivos, que frequentemente tem contraindicação ao uso de fármacos.

### 2) FA em pacientes com insuficiência cardíaca (IC):

A IC pode predispor um indivíduo à ocorrência de FA através de vários mecanismos, como o aumento da pressão de enchimento do ventrículo esquerdo ou a dilatação e a fibrose do AE, levando à remodelação estrutural e elétrica do átrio. A FA pode aumentar a mortalidade em pacientes com disfunção ventricular esquerda,^68^ portanto, o tratamento da FA nesse subconjunto de pacientes é de importância crucial,^69 - 73^ dadas as limitações da amiodarona, único medicamento antiarrítmico disponível para esse subgrupo. As mais recentes diretrizes europeias de 2020 referem indicação Classe IIa,^15^ com base em estudos comparativos com amiodarona (AATAC)^69^ e na publicação de estudos randomizados como o AMICA^74^ e CASTLE-AF,^75^ este último realizado em pacientes com IC grave (Fração de ejeção média 32%), demonstrando uma redução expressiva na mortalidade ou hospitalização por IC (38%) e na mortalidade cardiovascular (51%). Tais achados sem precedentes confirmam o prognóstico negativo da FA nessa população e abrem uma nova fronteira de indicações para ablação em centros com experiência e infraestrutura adequados. Resultados positivos recentes são animadores, com demonstração de melhora da função ventricular e reversão do remodelamento atrial.^76^

### 3) FA em idosos:

Há estudos que se concentraram em relatar os resultados da ablação da FA em indivíduos mais velhos. O limite de idade para a definição de idosos variou entre ≥70, 75 ou 80. O número de idosos nesses estudos foi, porém, pequeno, com cinco dos sete estudos inscrevendo menos de 100 pacientes, e os maiores resultados relatados em 261 idosos. Os resultados desses estudos fornecem evidências de que a ablação preenche critérios de segurança e eficácia nessa população,^77 , 78^ apesar de redução nas taxas de sobrevida livre de FA a cada década de idade (Classe IIa).

### 4) FA em assintomáticos e redução do risco de AVC:

Ablação da FA (paroxística ou persistente) em pacientes realmente assintomáticos pode ser considerada,^79^ a despeito das poucas evidências de significativa mudança em desfechos “duros” – em particular no risco de fenômenos tromboembólicos/AVC. Deve ser realizada por operador experiente e após uma discussão detalhada dos riscos e benefícios da realização do procedimento (Classe IIb). Há sólidas evidências de redução de hospitalizações^80^e uso de recursos, com relação custo-benefício favorável.^10^ Nesse cenário, deve-se priorizar pacientes com alta probabilidade de sucesso (jovens, FA paroxística, sem remodelamento atrial significativo).

Diversos estudos retrospectivos observacionais apontam para uma significativa redução do risco tromboembólico em pacientes com escore CHADSVASC ≤3 submetidos à ablação bem-sucedida,^81 - 87^ muitos deles reportando até desfechos favoráveis em pacientes que interromperam a terapia anticoagulante. Dados do estudo KP-RHYTHM^88^comprovando que a o risco de AVC é proporcional à carga de FA em pacientes paroxísticos, independentemente do escore CHADSVASC, e metánalise de estudos randomizados^89^ sugerindo redução de mortalidade e hospitalizações, são compatíveis com a hipótese de redução de risco após uma ablação bem-sucedida.

É preciso ressaltar, porém, que não há evidência direta de estudos randomizados especificamente desenhados para este objetivo; o estudo CABANA^7^ não demonstrou redução em desfechos combinados em uma população heterogênea (FA paroxística e persistente) submetida à ablação *versus* tratamento medicamentoso. O recém-publicado EAST-AFNET 4^90^ demonstrou significativo benefício em desfechos cardiovasculares com uma estratégia inicial visando ao controle do ritmo quando comparado com o controle da frequência cardíaca. Contudo, nesse importante estudo randomizado, apenas 20% dos pacientes foram tratados com ablação.

Por isso, todas as diretrizes atuais recomendam que o tratamento ablativo não tenha como objetivo a suspensão da terapia anticoagulante,^11 - 14^ que deve ter sua indicação a partir do risco de base do paciente (usualmente indicada em pacientes com escore CHADSVASC ≥2). Todos os pacientes submetidos à ablação devem ficar sob uso de anticoagulantes por período mínimo de 2 meses, independentemente dos fatores de risco, e sua continuação por período indeterminado deve ser individualizada pelo escore de risco.

O estudo OCEAN,^91^ em andamento, compara a manutenção da anticoagulação (rivaroxabana) com o ácido acetilsalicílico em pacientes de risco moderado a severo submetidos à ablação e mantendo ritmo sinusal por pelo menos 1 ano após o procedimento. Os resultados devem ajudar a refinar as indicações de anticoagulação a longo prazo pós-ablação.

## Complicações

O procedimento de ablação está associado a pequenas taxas de complicações em centros de excelência com grande volume e experiência, sendo as mais graves complicações individualmente menores que 1% nos centros com maior experiência.^11^ A Tabela 5 resume as principais complicações e suas incidências relatadas na literatura.

**Tabela 5:** Complicações relacionadas à ablação da fibrilação atrial

Complicações	Incidência	Teste diagnóstico
Morte	<0,1% a 0,4%	-
Estenose/oclusão de artéria coronária	<0,1%	Cineangiocoronariografia
Fístula atrioesofágica	0,02% a 0,11%	TC/RM; evitar endoscopia com insuflação de ar
Embolia aérea	<1%	Clínica ou angiografia
Estenose VP	<1%	TC/RM
Síndrome do AE duro	<1,5%	Eco; cateterismo cardíaco
Paralisia permanente do nervo frênico	0% a 0,4%	Raio X do tórax; fluoroscopia; *Sniff* test
AVC/AIT	0% a 2%	TC/RM; angiografia cerebral
Complicações vasculares	0,2% a 1,5%	Ultrassom vascular; TC
Tamponamento cardíaco	0,2% a 5%	Eco
Pericardite	0% a 5%	Clínica; ECG; Eco
Gastroparesia	0% a 17%	Endoscopia; deglutição de bário; estudo do esvaziamento gástrico

*TC: tomografia computadorizada; RM: ressonância magnética; VP: veia pulmonar; AE: átrio esquerdo; AVC: acidente vascular cerebral; AIT: ataque isquêmico transitório; ECG: eletrocardiograma.*

É importante estar atento a uma complicação tardia (nas primeiras semanas) relacionadas à lesão esofagiana devido à proximidade desse órgão com a parede posterior do AE. Durante a aplicação de energia nessa região, deve-se reduzir a potência e o tempo de aplicação, além do monitoramento da temperatura esofágica luminal ( Tabela 4 ). Uma alternativa já em uso corrente consiste em diversos modos de desvio do trajeto do esôfago, de forma a distanciá-lo do local de aplicação de RF.^92 - 95^ Há relatos de fístulas atrioesofágicas, com elevado índice de mortalidade.^96 - 100^ Felizmente, essa complicação tem incidência estimada ao redor de 0,04%, mas seu reconhecimento precoce pode ser fundamental para evitar um desfecho fatal.^99 , 101 - 103^

## Perspectivas futuras

O uso de alta potência de RF com curta duração ( *high power short duration* ) tem sido advogado como um modo de produzir lesões teciduais de melhor qualidade,^104 , 105^ além de provocar lesões mais largas e de menor profundidade e, portanto, com menor risco de danos colaterais (especialmente ao esôfago). Essa técnica foi associada a menor tempo de aplicações de RF e de instrumentação do átrio esquerdo e baixos índices de complicações,^106 , 107^ impulsionando novas investigações de cateteres que possam fazer lesões mais permanentes em poucos segundos de aplicação.^108^

Há uma grande expectativa com o desenvolvimento de uma nova modalidade de energia para ablação, denominada “eletroporação”. Diferentemente das energias térmicas (RF, crioterapia, *laser* , ultrassom e micro-ondas), cuja propensão é eliminar todos os tecidos indiscriminadamente, a ablação por campo pulsado ou “eletroporação” é uma modalidade ablativa não térmica, na qual os campos elétricos ultrarrápidos (<1s) são aplicados ao tecido-alvo de forma seletiva, desestabilizando as membranas celulares e culminando em morte celular. Isso é possível, pois os tecidos têm diferentes limiares para necrose. Essa tecnologia é usada para tratar tumores sólidos irressecáveis devido à proximidade de vasos sanguíneos ou nervos, dada a resistência a campos elétricos pulsados.^109 , 110^ Os cardiomiócitos têm um dos limiares de lesão tecidual mais baixos, podendo, por isso, ser aplicados limitando o dano colateral, como o esôfago^111^e o nervo frênico.^112^

Experiência inicial em pacientes submetidos a isolamento ultrarrápido das VP é bastante promissora, com índices de isolamento permanente nunca antes obtidos (100%).^113^ Essa tecnologia tem grande potencial para substituir definitivamente a RF e outras energias térmicas para tratamento da FA por cateter.

O estudo ERADICATE-AF,^114^ recentemente publicado, avaliou o efeito adicional da denervação renal por cateter em 302 pacientes hipertensos submetidos à ablação de FA, randomizados para isolamento das VP simples ou combinado à ablação na artéria renal. Nesse estudo, a adição da denervação resultou em melhor sobrevida livre de FA aos 12 meses (72% *vs.* 56%). Esses achados certamente necessitam ser replicados em um modelo cego ( *sham procedure* ) de denervação renal, porém, a modulação do sistema nervoso autônomo é um mecanismo fisiopatológico importante e que deve ser melhor explorado.

## Conclusão

A ablação por cateter é o método mais eficaz para controle do ritmo em pacientes com FA, associada à expressiva redução dos sintomas, da carga de FA e das internações hospitalares, com significativa melhora na qualidade de vida. É associada a baixas taxas de complicações quando realizada em centros experientes. Seu papel na redução de eventos tromboembólicos e na mortalidade ainda necessita de comprovação definitiva em futuros estudos randomizados, apesar desta forte tendência nos dados atualmente disponíveis.
