# Supplementary material for: Fibrilação Atrial (Parte 2) – Ablação por Cateter
Source: Arq Bras Cardiol. 2021 Feb 19;116(2):334–45. [Article in Portuguese] doi: 10.36660/abc.20200477 (PMC7909976; doi:10.36660/abc.20200477)
Supplement: Translation

# Translation: Atrial Fibrillation (Part 2) - Catheter Ablation

**Authors:** Eduardo B. Saad, Andre d’Avila

**Affiliations:** 1 Brazil Hospital Pró-Cardíaco - Serviço de Arritmias e Estimulação Cardíaca Artificial, Rio de Janeiro, RJ - Brazil; 2 Brazil Hospital Samaritano, Rio de Janeiro, RJ - Brazil; 3 Brazil Hospital SOS Cardio, Florianópolis, SC - Brazil; 4 USA Beth Israel Deaconess Hospital, Harvard Medical School, Boston - USA

**Keywords:** Arrhythmias Cardiac, Fibrilation Atrial, Catheter Ablation/methods, Echocardiography/methods

## Abstract

More than 20 years since its initial use, catheter ablation has become a routinely performed procedure for the treatment of patients with atrial fibrillation (AF). Initially based on the electrical isolation of pulmonary veins in patients with paroxysmal AF, subsequent advances in the understanding of pathophysiology led to additional techniques not only to achieve better results, but also to treat patients with persistent forms of arrhythmia, as well as patients with structural heart disease and heart failure.

Significant technological advances, especially in 3D electroanatomic mapping, intracardiac echocardiography use and how energy is delivered to the tissue (cryoablation and tissue contact force with radiofrequency) have allowed a significant reduction in the rate of complications and in the use of ionizing radiation.

Currently, ablation is the most efficient treatment for patients with AF, and an excellent alternative to the use of antiarrhythmic drugs, whose development has been insignificant in recent decades.

With the pioneering observations made by Haissaguerre et al.,^1^the pivotal role of arrhythmogenic foci located in the pulmonary veins (PV) in the pathophysiology of the initiation and maintenance of AF episodes was shown. The concept of focal AF was then established, where atrial arrhythmia that diffusely affects both atria have a well-determined origin, and is therefore susceptible to therapeutic interventions. Techniques using catheter ablation were developed and improved to eliminate AF-generating foci through circumferential ablation around the PVs,^2 - 4^with higher success and performance rates compared to the best pharmacological therapy.^5 - 10^

The aim of this article is to review the advances in catheter ablation for AF and describe to the clinical cardiologist state-of-the-art indications, techniques, results and complications.

## Ablation Strategies

Over the last 20 years, several ablation strategies have been used to control AF. In common, there is a current consensus that the isolation of all PVs is fundamental in all groups of patients (paroxysmal, persistent or long-standing persistent AF).^11 - 15^Isolation must be electrically proven by circular mapping inside the PVs (Figures 1 and 2), as this step is paramount for the success of the procedure. Recent studies have shown that the procedure should be performed on uninterrupted oral anticoagulation, a strategy proven to reduce thrombotic and hemorrhagic complications.^16 - 18^

In patients with paroxysmal AF, PV isolation is usually all that is needed, targeting additional sites only in specific situations (e.g., triggering foci mapped outside the PVs). Some centers routinely perform isolation of the superior vena cava^19 , 20^since it can also be, albeit rarely, *a* triggering AF-inducing source. Most publications show favorable results, with success rates above 70%.^6^

PV isolation can be performed using: 1) radiofrequency (RF) energy, through point-by-point focal applications ( Figure 1 – A), ideally with catheters with contact force sensors at the tip ( Figure 1 – B), or 2) freezing (cryoablation), using a balloon catheter positioned in the antrum of the PVs, capable of performing ablation simultaneously around the entire circumference in contact with the tissue ( Figure 1 - D). A randomized study (Fire and ICE)^21^directly comparing the two strategies for the treatment of paroxysmal AF showed similar results. These findings were replicated in a second randomized study (CIRCA-DOSE)^22^that compared two cryoablation regimens (4 min vs. 2 min freezings) to the use of contact force-guided RF to isolate the PVs in patients with paroxysmal AF; in this study, there was a >98% reduction in AF burden demonstrated through continuous electrocardiographic monitoring. It is important to note that the Cryo balloon catheter is not commonly used for ablation at sites other than around the PVs; when necessary, an RF catheter should be used for that ( Figure 1 - C).

**Figure 1:** – Catheter ablation for the treatment of paroxysmal AF. A) Isolation of left VPs by circumferential ablation (RF point-by-point) guided by 3D electroanatomic mapping (NAVx system — Abbott), demonstrating the elimination of electrograms (*) recorded by a circular catheter inside the PVs. B) Isolation of the right PVs (CARTO system — Biosense Webster) with a contact force-sensing catheter (shown by the force vector and force quantification = 7 g); the circular mapping catheter is inside the right superior PV. C) Persistent AF ablation (NAVx system — Abbott) demonstrating the additional linear RF lesions to isolate the LA posterior wall (roof and inferior lines), leading to the elimination of electrograms (recorded by the circular mapping catheter). D) Fluoroscopic imaging during cryoablation for isolation of the left superior PV, demonstrating the balloon catheter (arrow) inflated and in contact with the vein ostium. Balloon ablation along the PV circumference is performed simultaneously, which is usually restricted to PV isolation — when additional ablation is required, an RF catheter should be used.

In persistent and long-standing persistent forms of AF, additional electrical conduction barriers are often created, as stand-alone PV isolation is usually insufficient and associated with high recurrence rates.^23 - 25^Several strategies have been studied,^26 - 37^the most frequently used being: ablation of triggers outside the PVs, linear lesions in the left atrium (LA) and extensive RF applications at sites depicting fractionated electrograms during AF (most commonly observed in the posterior LA wall, septum, LA roof, mitral annulus, base of the left atrial appendage (LAA) and inside the coronary sinus). During RF applications at these sites, AF conversion to regular atrial tachycardias or even to sinus rhythm may occur.

The negative results of the randomized Star AF II^38^study should be noted. The study compared the addition of linear lesions and ablation of fragmented potentials to PV isolation in patients with persistent AF. In this study, there was no difference in the rates of sinus rhythm maintenance at 18 months between the groups (59% for PVI only vs. 49% and 46% in the other groups, without statistical significance). Therefore, many centers still perform PV isolation only, even in patients with persistent AF.

A more aggressive strategy for eliminating AF triggers was also tested in a randomized controlled trial (BELIEF Trial),^39^by electrical isolation of the left atrial appendage (LAA). Isolation of this structure in addition to conventional ablation was associated with a 55% reduction in the relative risk of AF recurrence in patients with long-term persistent AF. LAA isolation is currently performed selectively as it requires extensive RF applications and its association with increased risk of embolic phenomena (due to the loss of LAA contraction leading to slow flow and thrombus formation). Patients with electrically isolated LAA should be permanently anticoagulated, regardless of the CHADSVASC score, and should undergo occlusion of this structure if anticoagulation is contraindicated.^40^

Therefore, more persistent forms of AF with significant atrial remodeling require modification of the atrial substrate, implying a greater number, sites and extent of RF applications. There is no consensus in the literature on the best strategy to be used ( Table 1 ). The evolution of AF to persistent forms represent progression of a pathological process (atrial myopathy)^41 , 42^and should motivate earlier intervention, ideally when AF is still paroxysmal, and LA remodeling is not yet present. A large retrospective study with more than 4,500 patients analyzed the impact of time between the diagnosis of AF and ablation therapy.^43^The results are striking, demonstrating that the earlier the ablation is performed the better the results – establishing the so called “oncological concept of AF”, that is, the best results are obtained when treatment is done in the early stages of the disease (PV isolation in paroxysmal AF). In more advanced diseases (persistent and long-standing persistent AF), treatment is usually much more extensive and associated with worse results. A message to cardiologists and clinicians caring for AF patients is that the sooner the better.

**Table 1:** – Atrial fibrillation Ablation Strategies

For PV Isolation:	
Class I – A	PV isolation is recommended for all AF ablation procedures
Class I – B	Demonstration of PV entrance block
Class IIa – B	Monitor for PV reconnection for 20 minutes after initial isolation
Class IIb – B	Adenosine administration 20 minutes after PV isolation Pacing along the circumferential ablation line Demonstration of PV exit block
**In addition to PV isolation:**	
Class I – B	CTI ablation in patients with history of typical flutter or if the arrhythmia is inducible during AF ablation
Class I – C	If linear lesions are performed, bidirectional block should be demonstrated
Class IIa – C	If reproductible non-PV triggers are identified, ablation should be considered When using a contact force-sensor catheter, a minimum of 5-10 g is reasonable
Class IIb – B	LA posterior wall isolation can be considered for initial or redo procedures for persistent or long-standing persistent AF High dose Isoproterenol for non-PV trigger detection and ablation can be considered for initial or redo procedures for paroxysmal, persistent, or long-standing persistent AF

*PV: pulmonary vein; AF: atrial fibrillation; ICT: cavo-tricuspid isthmus; LA: left atrium.*

## Technologies to guide ablation

Regardless of the strategy used, imaging-based mapping methods are often used in addition to traditional electrophysiological mapping. Two types of technology are appropriate in this setting:

a) Electroanatomic mapping – this form of 3D mapping allows to accurately define the anatomy of the atrial cavities and the PVs, depict the functional substrate by measuring tissue voltage, mark the RF lesions spots ( figure 1 ) on the constructed map and color-code the electrical activation information obtained. It is also possible to navigate on images of the true anatomy obtained by computed tomography or magnetic resonance imaging. 3D mapping is especially useful to reduce exposure to fluoroscopy and to make easy to show electrical activation of the arrhythmia circuit or focus as well as the RF lesions performed to treat them. Two systems are currently available in Brazil: CARTO — Biosense Webster and NavX — Abbott.

b) Intracardiac echocardiogram (ICE) – through an ultrasound catheter initially positioned (but not limited to) in the right atrium, it is possible to obtain detailed real-time images of cardiac anatomy^44 , 45^and visualize precise and safe manipulation of catheters through the various cardiac structures ( Figure 2 ). Its use also allows the safe performance of transeptal punctures under direct visualization and the early detection of acute complications (pericardial effusion, thrombi). A recent study with more than 100,000 patients undergoing AF ablation showed the importance of this imaging method in significantly reducing the risk of a severe complication: cardiac perforation.^46^In this contemporary series, failure to use ICE was the greatest risk factor for cardiac perforation (RR 4.85).

**Figure 2:** Use of intracardiac echo (ICE) during AF ablation. A) Schematic diagram showing the ICE catheter in the right atrial cavity with the ultrasound beam directed to guide the two transeptal punctures and positioning of circular mapping and ablation catheters in LA. B) ICE image demonstrating PV antral positioning and tissue contact during RF delivery around the left superior PV (VPSE). LA: left atrium; LAA: left atrial appendage; Map: mapping catheter; RF: ablation catheter.

These non-fluoroscopic imaging tools have been increasingly used in the EP laboratory over the years and can even guide the entire ablation procedures, completely avoiding the use of X-rays.^47^Initially reported approximately 10 years ago, “Zero-Fluoro” techniques are increasingly used in the electrophysiological community because they have been shown as safe and effective as traditional methods guided by fluoroscopy.^48 - 50^

## Recurrences

Two main factors justify AF recurrences after ablation:

1. Reconnection or recurrent conduction in the PVs – for circumferential lesions to provide permanent PV isolation, contiguous fibrous tissue formation should form usually four to eight weeks after the acute injury (energy-induced tissue edema). If the lesion is not deep enough in the atrial wall, there may be remaining viable tissue after edema resorption. It only takes a small recovered segment to restore electrical PV-LA connection.

2. Occurrence of ectopic foci outside the PVs (non-PV triggers) – these occur more commonly (but not only) in persistent forms of AF or in patients with significant atrial remodeling.

PV reconnection is easily solved with new RF applications in conduction gaps. Reintervention is usually quick, easy and safe. In paroxysmal AF, it increases the control rates of AF in approximately 95% of cases. With the use of catheters with contact-force sensors, it has become an increasingly rare phenomenon^51 - 53^as RF lesions tend to be deeper and permanent.^54^

Non-PV triggers represent a more diffuse atrial substrate; their recognition and extensive ablation are necessary to improve outcomes, without which arrhythmia control is usually not possible.^33 , 54 , 55^They are most commonly located at the LA posterior wall, LAA and coronary sinus^32 , 54^— structures that can also be isolated by RF applications. It is certainly possible to maintain sinus rhythm in the long term, even if more than one intervention if necessary.

## Patient Selection and Results

The selection of patients for catheter ablation of AF is currently mainly based on the failure of medical therapy ( Table 2 ). According to the last HRS/EHRA/ECAS/APHRS/SOLAECE consensus of experts in 2017,^11^the primary indication for AF ablation is the presence of symptomatic paroxysmal or persistent AF, refractory or intolerant to at least one class I or III antiarrhythmic drug. There is solid evidence for improved quality-of-life parameters in these patients.^5 , 56^

**Table 2:** – Indications for atrial fibrillation ablation

Symptomatic AF, refractory or intolerant to at least 1 antiarrhythmic drug (class I or III):	
Class I – A	Paroxysmal AF
Class IIa – B	Persistent AF
Class IIb – C	Long-standing persistent AF
**Symptomatic AF, before initiation of antiarrhythmic drugs (Class I or III):**	
Class IIa – B	Paroxysmal AF
Class IIa – C	Persistent AF
Class IIb – C	Long-standing persistent AF
**Indications for patient populations underrepresented in clinical trials:**	
Class IIa – B	Congestive heart failure Older patients (≥ 75 years) Hypertrophic cardiomyopathy Younger patients (≤ 45 years) Brady-tachy syndrome
Class IIa – C	Athletes with AF
Class IIb – C	Asymptomatic AF

*AF: atrial fibrillation.*

AF ablation can be performed in patients with various types of heart disease (coronary artery disease, left ventricular hypertrophy, heart failure) and clinical presentations of AF (paroxysmal, persistent or long-lasting persistent), but the best results are obtained for patients with structurally normal hearts. In the largest randomized study that compared ablation with pharmacological therapy (CABANA),^7^survival free of recurrent AF is significantly better (HR 0.53) in ablated patients compared to those who remained on multiple antiarrhythmic drugs. Nevertheless, in this study, there was no reduction in a combined hard endpoint (death, stroke, severe bleeding or cardiac arrest) in the “intention-to-treat” analysis, although there were problems with large crossover rates for the ablation group (27%). In this study, the subgroups that benefited the most were the youngest (<65 years) and patients with congestive heart failure.

The selection of patients with persistent and long-standing persistent forms of AF follows the same reasoning, but the decision should be individualized according to parameters of remodeling such as LA size or volume^57^(which is an important predictor of recurrence) and AF duration. Persistent AF is a heterogeneous disease, with different degrees of atrial fibrosis and with influence of the autonomic nervous system and other pathophysiological processes still poorly understood, which explains the heterogeneous results observed with different ablation strategies. Targeting this type of AF requires an individualized definition of the substrate and mechanisms involved.^58 , 59^

It is important to note that even with the strategy of extensive RF applications described above, higher recurrences rates and need for reinterventions are observed. In the experience of Natale et. al., 60% of patients maintained sinus rhythm without drugs after the first procedure.^54^In those undergoing a second intervention, 80% maintained sinus rhythm. Table 3 summarizes some of the main published studies.

**Table 3:** – Trials in atrial fibrillation ablation

Trial (year)	Type	Ablation Strategy	N	Follow-up months	Sinus Rhythm Maintenance	p-value
**Paroxysmal AF**						
Thermocool AF (2010)	Randomized Ablation or AAD; multicentric	PVI CFAE and lines optional	167	12	66%/16%	<0.001
STOP AF (2013)	Randomized Cryoablation or AAD; multicentric	PVI	245	12	70%/7%	<0.001
SMART AF (2014)	Non-randomized; contact force-sensors; multicentric;	PVI CFAE and lines optional	172	12	72.5%/NA	<0.0001
TOCCASTAR (2015)	Randomized contact force-sensors or not; multicentric	PVI CFAE, non-PV triggers and lines optional	300	12	67.8%/69.4%	0.0073*
RAAFT-2 (2014)	Randomized Ablation or AAD (1^st^line); multicentric	PVI non-PV triggers optional	127	24	45%/28%	0.02
MANTRA-PAF (2012)	Randomized Ablation or AAD (1^st^line); multicentric	PVI + roof lines mitral and ICT lines optional	294	24	AF burden: 13%/19%	-
FIRE and ICE (2016)	Randomized RF or Cryo; multicentric	PVI	762	12	64.1% (RF)/65.4% (Cryo)	-
CIRCA DOSE (2019)	Randomized RF or Cryo 4 min or Cryo 2 min; multicentric	PVI	346	12	53.9% (RF)/52.2% (Cryo 4 min)/51.7% (Cryo 2 min)	0.87
**Persistent AF**						
TTOP (2014)	Randomized Ablation or AAD / DCC	PVI + CFAE	210	6	56%/26%	< 0.001
SARA (2014)	Randomized Ablation or AAD; multicentric	PVI CFAE and lines optional	146	12	70%/44%	0.002
STAR AF II (2015)	Randomized 3 ablation strategies; multicentric	PVI; PVI + CFAE; PVI + lines	589	18	59%/49%/46%	0.15
**Paroxysmal or Persistent AF**						
CABANA (2019)	Randomized Ablation or AAD; multicentric	PVI additional ablation optional	2204	48.5	69%/50%	-

AF: atrial fibrillation; RF: radiofrequency; AAD: antiarrhythmic drugs; PVI: pulmonary vein isolation; CFAE: complex fractionated atrial electrograms; DCC: direct current cardioversion; * non-inferiority.

Catheter ablation is less effective in certain subgroups of patients,^60^where advances in pathophysiological knowledge are still needed: dilated and fibrous atria, persistent or long-standing AF, hypertrophic cardiomyopathy, amyloid infiltrate, obesity and sleep apnea.

Long-term follow-up of patients undergoing catheter ablation shows the occurrence of late recurrences,^61 - 63^around 7% per year in the first 5 years. It should be noted that the estimation of the actual success of ablation is hampered by inconsistencies and heterogeneities in the definitions of success and recurrences in the different published studies. As an example, most studies consider as a recurrence any atrial arrhythmia lasting more than 30 seconds, a definition with clearly little clinical significance. In this scenario, the AF burden should be more valued and clinically meaningful in future research.

As new technologies and experiences tend to promote permanent PV isolation, recurrence is currently more frequently observed due to the appearance of non-PV triggers, which should be identified and addressed^32 , 33 , 54 , 64^. Therefore, it is important to maintain periodic monitoring of patients and it is prudent to maintain anticoagulant therapy in patients at higher risk who do not have contraindications.

Table 4 summarizes adjuvant care to maximize the safety and efficacy of the ablation procedure.

**Table 4:** – Adjunctive Strategies for atrial fibrillation ablation

Not directly related to the AF ablation procedure:	
Class IIa – B	Weight loss Evaluate BMI for ablation procedure Screen for sleep apnea signs and symptoms Treat sleep apnea
Class IIb – C	Interruption of AAD before ablation to improve long-term results is not clear AAD use during blanking period (3 months) after ablation to improve results is not clear
**Reducing risk during ablation procedure:**	
**Class I – B**	**Clear delineation of PV ostia to avoid energy delivery inside the PVs**
Class I – C	Reduce the energy power delivered in the LA posterior wall near the esophagus
Class IIa – C	Use a temperature sensor probe in the esophageal lumen and guide energy titration

*AF: atrial fibrillation; AAD: antiarrhythmic drugs; BMI: body mass index; PV: PV: pulmonary vein; LA: left atrium.*

## Special Situations

International guidelines published in 2016 and 2017 and updated in 2019 and 2020 by different international societies (SBC/HRS/EHRA/ECAS/APHRS/ACC/AHA/ESC/EHRA)^11 - 13 , 15^almost consensually recommend ablative treatment in special situations ( Table 2 ):

### 1) Ablation as first-choice therapy:

Increasing safety and efficacy allow ablation to be offered as first-line therapy for treatment (even before the use of antiarrhythmic drugs) in some special situations (athletes, young people, normal hearts).^65 , 66^It is a Class IIa indication for patients with symptomatic paroxysmal or persistent AF. Other appropriate situations for this strategy are patients with symptomatic pauses upon arrhythmia interruption (brady-tachy syndrome)^67^or in competitive athletes, who may have contraindications to antiarrhythmic drug use.

### 2) AF in patients with Heart Failure (HF):

HF may predispose to AF occurrence through various mechanisms, such as increased left ventricular filling pressures or LA dilatation and fibrosis, leading to atrial structural and electrical remodeling. AF can increase mortality in patients with left ventricular dysfunction.^68^Treatment of AF in this subset of patients is of critical importance^69 - 73^given the limitations of Amiodarone, the only antiarrhythmic drug available for this subgroup. In the most recent European guidelines published in 2020, AF ablation in patients with HF received a Class Ia^15^indication based on comparative studies with Amiodarone (AATAC)^69^and the publication of randomized studies such as AMICA^74^and CASTLE-AF,^75^the latter performed in patients with severe HF (mean EF 32%), demonstrating a significant reduction in mortality or hospitalization for HF (38%) and cardiovascular mortality (51%). These unprecedented findings confirm the negative prognosis of AF in this population and open a new frontier of indications for ablation in centers with adequate experience and infrastructure. Recent positive results are encouraging, with demonstration of improvement in ventricular function and reversal of atrial remodeling.^76^

### 3) AF in the elderly:

There are studies that have focused on reporting the results of AF ablation in older individuals. The age limit for the definition of elderly ranged from ≥70, 75 or 80 years. However, the number of elderlies in these studies was relatively small, with five of the seven studies enrolling less than 100 patients and the largest series reporting on 261 patients. Overall, the results of these studies provide evidence that ablation meets safety and efficacy criteria in this population,^77 , 78^despite a reduction in AF-free survival rates with every decade of age (Class IIa).

### 4) AF in asymptomatic patients and reduced risk of stroke:

Ablation of AF (paroxysmal or persistent) in truly asymptomatic patients can be considered^79^despite the lack of definitive evidence of significant changes in hard outcomes – particularly in the risk of thromboembolic phenomena/stroke. It should be performed by experienced operators and after a detailed discussion of the risks and benefits (Class IIb). There is solid evidence of reduction of hospitalizations^80^and resource utilization, with favorable cost-effectiveness.^10^In this scenario, patients with a higher probability of success should be prioritized (young people, paroxysmal AF, without significant atrial remodeling).

Several retrospective observational studies point to a significant reduction in thromboembolic risks in patients with CHADVASC score ≤3 undergoing successful AF ablation,^81 - 87^many of them reporting favorable outcomes even in patients who discontinued anticoagulant therapy. Data from the KP-RHYTHM^88^study, proving that the risk of stroke is proportional to the burden of AF in paroxysmal patients, regardless of CHADVASC score, and a metanalysis from randomized studies^89^suggesting reduced mortality and hospitalizations, are compatible with the hypothesis of risk reduction after a successful ablation.

It should be emphasized, however, that there is no direct evidence from randomized studies specifically designed for this purpose; the CABANA^7^trial did not show any reduction in a combined endpoint in a heterogeneous population (paroxysmal and persistent AF) comparing ablation versus drug treatment. The recently published EAST-AFNET 4^90^demonstrated a significant benefit in cardiovascular outcomes with a strategy of early rhythm control compared to heart rate control, but in this important randomized study, only 20% of patients were treated with ablation.

All current guidelines recommend that ablative treatment should not aim at discontinuation of anticoagulant therapy,^11 - 14^which should have its indication based on the baseline risk of the patient (usually indicated in patients with CHADSVASC score ≥ 2). All patients undergoing ablation should use anticoagulants for a minimum period of 2 months regardless of risk factors, and its continuation should be individualized by the risk score.

The ongoing OCEAN^91^study compares the maintenance of anticoagulation therapy (Rivaroxaban) with Aspirin in patients at moderate to severe risk undergoing ablation and maintaining sinus rhythm for at least 1 year after the procedure. The results should help refine indications of long-term anticoagulation after ablation.

## Complications

The ablation procedure is associated with low complication rates in centers of excellence with high volume and experience, with major complications individually lower than 1% in highly experienced centers.^11^ Table 5 summarizes the main complications and their incidences as reported in the literature.

**Table 5:** – Complications Related to AF Ablation

Complications	Incidence	Diagnostic test
Death	<0.1% – 0.4%	-
Coronary stenosis / occlusion	<0.1%	Coronary angiogram
Atrio-esophageal fistula	0.02% – 0.11%	CT/MRI; avoid endoscopy with air insufflation
Air embolism	<1%	Clinical or angiography
PV stenosis	<1%	CT/MRI
Stiff LA syndrome	<1.5%	Echo; cardiac catheterization
Permanent phrenic nerve paralysis	0% – 0.4%	Chest X-Ray; fluoroscopy; Sniff test
Stroke/TIA	0% – 2%	CT/MRI; cerebral angiography
Vascular complications	0.2 – 1.5%	Vascular ultrasound; CT
Cardiac tamponade	0.2% – 5%	Echo
Pericarditis	0% – 5%	Clinical; ECG; Echo
Gastroparesis	0% – 17%	Endoscopy; barium swallow; gastric emptying evaluation

*CT: computed tomography; MRI: magnetic resonance; PVI: pulmonary vein; LA: left atrium; TIA: transient ischemic attack; ECG: electrocardiogram.*

It is important to be aware of a late complication (in the first weeks) related to esophageal injury due to its proximity to the LA posterior wall. During energy application in this region, power and/or time should be reduced, in addition to monitoring the luminal esophageal temperature ( Table 4 ). An available alternative consists of different methods of mechanical esophageal deviation to increase its distance from the site of energy delivery.^92 - 95^There are reports of atrio-esophageal fistulas, with a high mortality rate.^96 - 100^Fortunately, this is a rare complication, with an estimated incidence of approximately 0.04%. Its early recognition is critical to avoid a fatal outcome.^99 , 101 - 103^

## Future Perspectives

The use of high-power RF with short duration has been advocated to produce better quality tissue lesions,^104 , 105^besides causing wider and shallower lesions and therefore less risk of collateral damage (especially to the esophagus). This technique was associated with shorter RF application and LA instrumentation times and low complication rates,^106 , 107^boosting further investigations of catheters that can cause more permanent lesions within seconds of energy delivery.^108^

There are great expectations for the development of a new energy source for ablation: “electroporation”. Unlike thermal energies (RF, cryotherapy, laser, ultrasound and microwave), which damages all tissues indiscriminately, pulsed field ablation (PFA) or “electroporation,” which is a non-thermal ablation modality in which ultrafast electric fields (<1 s) are applied to target tissue selectively, destabilizing cell membranes and culminating in cell death. This is possible because tissues have different thresholds for necrosis. This technology is already in use to treat unresectable solid tumors in close proximity to blood vessels or nerves, given their different resistance to pulsed electric fields.^109 , 110^Cardiomyocytes have one of the lowest tissue injury thresholds, and PFA can therefore be applied during catheter ablation, limiting collateral damage to nearby structures such as the esophagus^111^and phrenic nerve.^112^

Initial experience in patients undergoing ultra-fast PV isolation is very promising, with permanent isolation rates never reported before (100%).^113^This technology has great potential to replace RF and other thermal energies for catheter treatment of AF.

The recently published ERADICATE-AF^114^study evaluated the additional effect of catheter renal denervation in 302 hypertensive patients undergoing AF ablation, randomized to simply PV isolation or combined with renal artery ablation. The addition of denervation resulted in better AF-free survival at 12 months (72% vs. 56%). These findings certainly need to be replicated in a blinded model of renal denervation, but modulation of the autonomic nervous system is an important pathophysiological mechanism that should be further explored.

## Conclusions

Catheter ablation is the most effective method for rhythm control in patients with AF, associated with significant improvement in symptoms, AF burden, quality of life and hospital admissions. It is associated with low complication rates when performed in experienced centers. Its role in reducing thromboembolic events and mortality still needs definitive proof in future randomized studies.
